# Time to Total Knee Arthroplasty after Intra-Articular Hyaluronic Acid or Platelet-Rich Plasma Injections: A Systematic Literature Review and Meta-Analysis

**DOI:** 10.3390/jcm11143985

**Published:** 2022-07-09

**Authors:** Sabryne Berkani, Alice Courties, Florent Eymard, Augustin Latourte, Pascal Richette, Francis Berenbaum, Jérémie Sellam, Karine Louati

**Affiliations:** 1 Rheumatology Department, Inserm UMRS_938, (AP-HP) Saint-Antoine Hospital, Sorbonne Université, 75012 Paris, France; sabryne.berkani@aphp.fr (S.B.); alice.courties@aphp.fr (A.C.); francis.berenbaum@aphp.fr (F.B.); 2 Rheumatology Department, AP-HP Henri Mondor Hospital, 94000 Créteil, France; florent.eymard@aphp.fr; 3 Rheumatology Department, Inserm U1132, DMU Locomotion, AP-HP Lariboisière Hospital, Université de Paris, 75010 Paris, France; augustin.latourte@aphp.fr (A.L.); pascal.richette@aphp.fr (P.R.)

**Keywords:** knee osteoarthritis, hyaluronic acid, platelet-rich plasma, total knee arthroplasty, treatment

## Abstract

Intra-articular (IA) hyaluronic acid (HA) and platelet-rich plasma (PRP) injections are increasingly being prescribed for knee osteoarthritis (KOA). However, failure of the medical treatment may result in total knee arthroplasty (TKA). We wondered if IA HA or PRP injections (intervention) may delay the time to TKA (outcome) among KOA patients (population), compared to KOA patients not receiving these injections (comparator). For this systematic literature review (SLR) and meta-analysis, we selected observational studies with at least one group of patients receiving IA HA or PRP and with TKA data available. The main outcome was time from the diagnosis of KOA to TKA. We included 25 articles in the SLR (2,824,401 patients) and four in the meta-analysis. The mean strengthening the reporting of observational studies in epidemiology (STROBE) score was 63%. For patients receiving versus not receiving HA injections, the delay between a declared diagnosis of KOA to TKA was increased by 9.8 months (95% CI (8.2–11.4)). As compared with standard of care, the effect size of HA injections for this outcome was 0.57 (95% CI (0.36–0.76)). Only one study described a median time from PRP injections to TKA of 4.1 years (range 0.3–14.7). IA HA injections were associated with increased time to TKA. Causality cannot be concluded because of missing confounder factors as comorbidities. Data were insufficient to conclude any effect of PRP injections on TKA delay.

## 1. Introduction

Osteoarthritis (OA) is the most prevalent chronic joint disease, causing pain, disability and progressive joint destruction. Due to the sedentariness caused by the difficulty to walk, knee OA (KOA) is associated with mortality and cardiovascular diseases [1,2,3,4]. It is responsible for a substantial economic burden [5].

The management of OA is only symptomatic and should be individualized for each patient. It may use non-pharmacological (physiotherapy, exercise training, knee braces, etc.) [6,7] and pharmacological options (analgesic, non-steroidal anti-inflammatory, intra-articular [IA] injections of corticosteroids) [8,9]. Among pharmacological options, IA hyaluronic acid (HA) injections have been used for several years for symptom reduction, although controversial [10]. IA HA injections are recommended in the guidelines of the Osteoarthritis Research Society International (OARSI) and the French Society of Rheumatology [8,11], but conditionally not in the guidelines of the American College of Rheumatology (ACR) and the American Academy of Orthopaedic Surgeons [9,12].

The mechanism of action of HA injections remains unclear. IA HA injections may replace endogenous HA, stimulate endogenous HA synthesis by fibroblasts and inhibit cartilage degradation by aggrecanase [13,14,15]. The structural effects of HA on OA progression have not been demonstrated on X-ray images [16,17], but some studies have suggested a protective effect on cartilage by slowing down the cartilage volume loss and the aggravation of the cartilage defect score as compared with controls on magnetic resonance imaging (MRI) [18].

IA platelet-rich plasma (PRP) injections, which are increasingly being used in KOA [19], are based on the ability of platelets to release growth factors and many other beneficial mediators [20]. PRP injections may reduce pain and disability as compared with placebo [21,22], but their clinical efficacy has been challenged by recent negative studies [23]. The ACR and OARSI guidelines strongly recommend against their use because of lack of high-quality trials and lack of standardized preparation of PRP [9,11]. In a few studies, PRP injections have had positive effects on cartilage in animal models and slowed OA [24]. The growth factors released after platelet activation, including vascular endothelial growth factor, transforming growth factor beta and platelet-derived growth factor, may stimulate chondrocyte proliferation and mesenchymal stem cell differentiation and inhibit NF-κB [20,25,26].

Finally, total knee arthroplasty (TKA) is proposed in cases of failure of optimal well-conducted medical treatment. However, TKA may be responsible for serious adverse events (e.g., thrombosis or infection). Moreover, about 20% of patients continue to have chronic pain despite TKA [27].

As OA is a slowly progressive disease, long-term efficacy is difficult to assess. The outcome measures used in randomized clinical trials (RCTs) to estimate the efficacy of medical treatment in OA are symptoms (i.e., pain and function) and structural outcomes (by X-ray images or MRI). TKA has been recently considered one of the most clinically relevant hard endpoints for assessing the relevance of any knee OA treatment [28,29]. In one RCT with a small number of patients, more TKAs were performed in the placebo than HA group (OR = 0.41 95% CI (0.09–1.89)) [30]. Another RCT suggested that HA injection may delay the time to TKA but without reaching significance, with a mean difference in delay from IA HA injection to TKA of 3.8 months (*p* = 0.219) between the HA and placebo groups. However, such an endpoint requires several years of follow-up, which limits its use in pivotal trials.

Considering the possible protective effects on cartilage and long-term symptomatic efficacy of IA HA or PRP injections, we aimed to determine whether these treatments could increase the time to TKA in OA patients. For this, we performed a systematic literature review and meta-analysis of observational studies with the requirement of TKA as an outcome to investigate whether IA HA and PRP injections are associated with a delay in TKA.

## 2. Materials and Methods

### 2.1. Systematic Literature Search

We systematically reviewed the literature according to the Cochrane guidelines [31] and the Preferred Reporting Items for Systematic Reviews and Meta-Analysis (PRISMA) checklist [32]. Relevant publications were selected from PubMed and Embase with no limit on time (up to 28 January 2022) or language. The Cochrane database was not consulted because it does not include observational studies. The systematic review was registered on PROSPERO (CRD42020166804).

The keywords for the PubMed research were (“OA, Knee” [Mesh]) AND (“Hyaluronic acid” OR “Viscosupplementation” OR “Viscosupplements” OR “Platelet-Rich Plasma” OR “Blood Platelets” [Mesh]) AND (“OA, Knee/surgery” [Mesh] OR “Arthroplasty, Replacement, Knee” [Mesh] OR “Knee Prosthesis” [Mesh] OR “KA” OR “Knee replacement”).

We also searched abstracts for the past 2 years (January 2020 to January 2022) from meetings of the ACR, European League Against Rheumatism, French Society of Rheumatology, American Academy of Orthopedic Surgeons, Société Française de Chirurgie Orthopédique et Traumatologique (SOFCOT), American Association of Hip and Knee Surgeons and OARSI. The search was completed by manual research of included studies and direct data from the French Knee and hip osteoarthritis long term assessment (KHOALA) cohort [33].

### 2.2. Study Selection

To assess the effect of IA HA or PRP injection (intervention) on TKA delay (outcome) among patients with KOA (population) compared to KOA patients not receiving these injections (comparator), French and English observational (cohort, case–control and cross-sectional) studies of humans were selected, with the following criteria: at least one group of patients had received IA HA or PRP injections for KOA, with TKA as an outcome. To compare IA injection and non-IA injection groups, three variables of interest were determined in both groups: time from diagnosis of KOA to TKA (in month or year), time from IA HA or PRP injection to TKA (in month or year) and prevalence of TKA. We excluded studies with IA injections performed during TKA surgery and interventional studies. Two independent readers (SB and KL) selected articles based on titles and abstracts, then on full texts. In case of doubt, JS has been consulted and KL performed the final decision.

### 2.3. Data Extraction

The participants were separated into two groups: patients who received IA injections and patients who did not. We extracted the following data: publication data (title of the article, first and last author, publication date), study design (type of study, year(s) of inclusion, name of the database of inclusion, study quality score), population (total number of patients included, mean age and sex of patients, associated comorbidities), data on KOA (Kellgren–Lawrence (KL) grade, visual analog scale (VAS) for pain), type of HA (brand name, number of courses), method to obtain PRP and data needed for statistical analysis (time from a declared diagnosis of KOA to TKA or from IA injection to TKA (mean, standard deviation [SD]; median (interquartile range [IQR]) or number of TKAs during the observation time).

The quality of publications was assessed by the strengthening the reporting of observational studies in epidemiology (STROBE) quality score for observational studies (total score 22 points, expressed in percentage) [34,35].

### 2.4. Statistical Analysis

To examine the delay to TKA, the primary outcome was time from a declared diagnosis of KOA to TKA. The secondary outcomes were time from IA HA or PRP injection to TKA and the prevalence of TKA at fixed endpoints from a declared diagnosis of KOA or from the first IA injection mentioned in the study. We performed separate analyses for HA and PRP injections.

For each outcome, the results were analyzed descriptively for each study with calculation of mean (+SD) and/or median (+IQR) time and/or mean prevalence (%). The outcomes were also described for each HA course when data were available. Then, for the primary outcome when we had enough data, we performed a pooled analysis to evaluate the association between IA injection and time to TKA: for continuous variables, we calculated an effect size (ES) by the Hedges’ formulations adjusted *g*, for IA HA products compared to a control group. The ES is the standard mean deviation: mean difference/standard deviation at baseline. If the ES is <0.2, the effect of treatment is considered trivial, >0.2 to 0.5 small, >0.5 to 0.8 moderate, >0.8 to 1.2 high and >1.2 very high [36]. For binary variables, we calculated the odds ratio (OR). The accuracy of the ES and the OR is expressed by the 95% confidence interval (CI). We pooled analyses for HA but not PRP injections because of a lack of available data.

We used Revman V.5.3 for the meta-analysis. Heterogeneity was assessed by the I² index; with I² > 50% (high heterogeneity), we used a random-effects model, and with I² < 50% (low heterogeneity), a fixed-effects model.

## 3. Results

### 3.1. Literature Search and Characteristics of Included Studies

**Figure 1:**
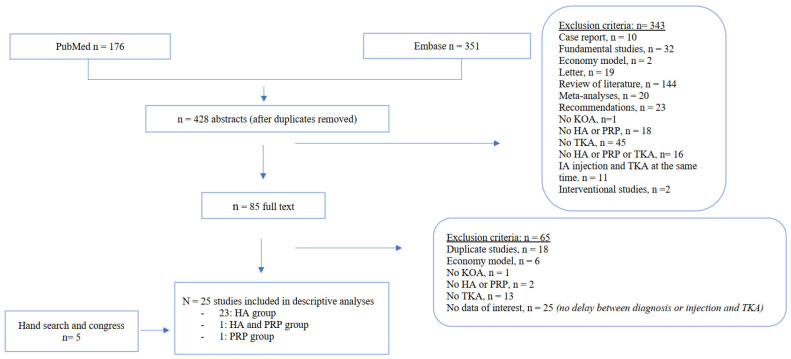
Flow chart of included studies. HA = hyaluronic acid; TKA = total knee arthroplasty; KOA = knee osteoarthritis, PRP = platelet-rich plasma.

A total of 25 studies met the selection criteria and were included in the descriptive analysis (Figure 1): 23 of HA injections, 1 of PRP injections and 1 of HA or PRP injections. The studies were from the United States [37,38,39,40,41,42,43,44,45,46,47,48,49,50,51,52], Spain [53,54], France [55,56,57], Turkey [58], Thailand [59], Finland [60] and Australia [61]. The mean STROBE quality score was 63% [34,35], but the results were heterogeneous (I² = 99%). All the studies were retrospective except three of them [45,56,57]. Details about the studies are in Table 1.

Ten studies were included for the primary outcome (9 retrospective [37,38,39,40,41,42,50,51,55] and 1 prospective [57]). The data from KHOALA cohort were obtained for an observational study [57] and directly from the cohort database [33].

### 3.2. Patient and Product Characteristics

In total, 2,824,401 patients were included; the mean age was 55.1 ± 12.8 years [58] to 70.5 ± 9.2 years [47] and the mean prevalence of women was 49.3% [47] to 83% [58]. In studies using a database, patients with a diagnosis of KOA were identified according to the International Classification of Diseases, 9th revision (ICD-9) or ICD-10 codes, and the comparison involved those receiving or not receiving IA HA or PRP injections [37,38,39,40,41,42,43,50,51,55]. In the other studies, in addition to the KOA diagnosis, IA injection was an inclusion criterion [43,44,45,46,47,48,49,52,53,56,58,59,60,61,62]. Pain VAS score, KL score and clinical severity were heterogeneous among studies.

The characteristics of studies and patients are in Table 2. Concerning comorbidities (which may influence the decision of knee joint replacement due to perioperative risk), in the study of Delbarre et al., the Charlson comorbidity index (CCI) did not differ between patients receiving and not receiving HA injections [55]. In the study by Altman et al., patients in the HA group were younger and the mean CCI was lower than in the other group (0.6 vs. 0.7, *p* < 0.001) [38]. In another study, the prevalence of comorbidities was 61.6% in the HA group versus 33% in the PRP group [60]. Some studies mentioned body mass index (BMI): in the study by Annaniemi et al., the frequencies of obesity were 67% and 45.3% in the PRP and HA groups, respectively. However, again, there was no group without any treatment [60]. The other studies mentioning BMI did not have a control group. The injection products used and their controls are in Supplementary Materials (Table S1).

### 3.3. Primary Outcome: Time from KOA Declared Diagnosis in the Database to TKA (HA Injection)

#### 3.3.1. Descriptive Delay from Each Study

In the 10 studies included [37,38,39,40,41,42,50,51,55,57], 418,266 patients underwent TKA (Table 2); 142,210 (34%) had received HA injections.

In seven studies giving appropriate data for such analyses [37,38,39,40,42,50,55], the median time from a KOA diagnosis to TKA was significantly longer for patients receiving (range 15.8 months [38] to 29.8 months [37]) than not receiving HA injections (3.7 [38] to 12 months [40,42,55], *p* < 0.001), and the mean time was significantly longer for patients receiving injections (19.7 ± 14.2 [38] to 39.4 months (SD unknown) [42] vs. 8.9 ± 11.6 [38] to 27.4 months (SD unknown) [42]). Only in the French cohort KHOALA did the mean time from a declared diagnosis of KOA to TKA not significantly differ between the HA injection and control group: 9.67 ± 6.77 and 10.56 ± 9.14 years (*p* = 0.58) [57].

#### 3.3.2. Pooled Mean Delay and ES

**Figure 2:**
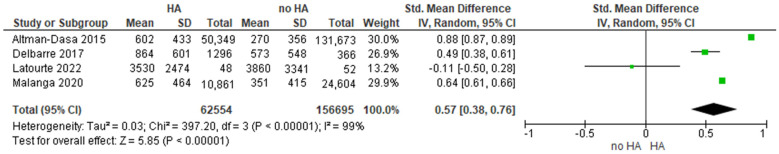
Forest plot of effect of IA HA injection against time from KOA declared diagnosis in the database to TKA. HA = hyaluronic acid, SD = standard deviation, Std mean difference = standard mean difference, 95% CI = confidence interval.

In the pooled analyses, we excluded six articles (mean time unavailable [40,51], lacking SD [37,39,42] or from redundant databases [39]). Then, for four observational studies, the mean time from a declared diagnosis of KOA to TKA for patients receiving than not receiving HA injections was 614 ± 456 days (20 months) versus 234 ± 377 days (7.7 months). The mean difference was 299 days (95% CI (250–348)) (i.e., 9.8 months (95% CI (8.2–11.4)) between the two groups and the ES for HA injections was 0.57 (95% CI (0.38–0.76)) (Figure 2) [38,50,55,57].

#### 3.3.3. Multiple Courses of HA Injections

The mean and median times to TKA increased with each additional HA session in two studies [37,38]. The difference in median time between one and two courses was 262 or 409 days in two different studies, and between three and four courses was 227 or 317 days [37,38]. Additionally, in the study of Abott et al., each treatment increased the median gap by 202 days on average [51]. In the study of Ong et al., the median time to TKA increased from approximately 20 months for one course to almost 5 years for five or more courses [40].

### 3.4. Secondary Outcomes: Time from IA Injection to TKA (HA Injection)

Seven studies mentioned time from IA injection to TKA [43,44,45,50,52,53,59], ranging from 6.7 [52] to almost 36 months [53]. The pooled mean time from IA HA injection to TKA was 14.3 ± 11.1 months [43,44,45,50,52,59]. Only one study (*n* = 20) had a control group: TKA was 14 months later for patients who received than did not receive HA injections (*p* < 0.001) [53].

### 3.5. Secondary Outcomes: Prevalence of TKA at 2 Years after a Declared Diagnosis of KOA

From the results of two studies with a total of 2,045,279 patients, the prevalence of TKA at 2 years after declared diagnosis of KOA in the database did not differ between patients receiving or not receiving HA injections: OR = 3.07 (95% CI (0.60–15.74)) [41,55].

### 3.6. Secondary Outcomes: Prevalence of TKA at Different Times after IA HA Injection

We extracted the reported prevalence of TKA after IA HA injections (Table 3). At 1 year after IA HA injection, the mean TKA prevalence was 5.2% (95% CI 3.7–6.7]) for 877 patients [47,58,62] and at 2 years was 8.2% (95% CI (5.8–10.6)) for 520 patients [47,62].

### 3.7. PRP Injections

We included two studies with a group of patients who underwent IA PRP injections [54,60], but only one had a control group [60]. The characteristics of patients are in Table 2. We extracted the prevalence of TKA after PRP injections (Table 3).

Annaniemi et al. retrospectively compared patients who received HA or PRP injections, with a mean follow-up of 17 months after PRP injections. The groups were not comparable at baseline: as compared with the HA group, the PRP group was younger, the prevalence of comorbidities and diabetes was lower and the prevalence of obesity was higher. During follow-up, 5 patients in the PRP group and 31 in the HA group underwent TKA (*p* < 0.001) [60].

The study of Sanchez et al. was an observational retrospective cohort of patients receiving PRP injections, with no control group. The median time from IA PRP injection to TKA was 4.1 years (range 0.3–14.7) for 186 patients [54].

## 4. Discussion

This study aimed to determine whether IA HA or PRP injections may delay the requirement for TKA for patients with KOA. In studies using a database, the median and mean times from diagnosis of KOA to TKA were significantly longer for patients receiving IA HA injections: TKA occurred almost 10 months later for HA than no-HA injection groups. The ES for IA HA injections from KOA declared diagnosis in the database to TKA compared to usual care was moderate (0.57) [38,50,55,57]. The pooled mean time from IA HA injection to TKA was 14.3 ± 11.1 months [43,44,45,50,59]. For this analysis, only one study had a control group. For PRP injections, only one study described a median time from PRP injection to TKA of 4.1 years (range 0.3–14.7) but without any comparator [54].

The time from IA HA injection to TKA may be considered “earned time” without TKA. Delaying TKA by 1 year seems achievable with HA injections. However, it does not necessarily reflect a structural effect of HA. Indeed, a treatment may delay TKA by reducing the pain [55,63,64]. Miller et al. showed a 20% reduction in VAS knee pain with intra-articular sodium hyaluronate injection in most patients who chose to delay TKA [47]. HA could allow for modulating the time for the TKA decision according to the patient’s needs, for example, to manage a comorbidity that may interfere with the surgery, to lose body mass, to start a muscular strengthening program against sarcopenia or to align with the personal will.

Delaying a TKA is cost-effective. Ong et al. followed patients for 2 years after KOA diagnosis according to the database and calculated healthcare costs. In all, 96.8% of patients did not undergo TKA, but 69% of the healthcare costs were attributed to surgical care in the United States. The total savings for patients who received IA HA injections and did not undergo TKA would have been up to US 1.84 billion dollars [41]. In another study, HA injections represented only a small fraction (3%) of the overall costs [65].

These results should be interpreted with caution because the common slow evolution of KOA can lead to repeated HA injections. In other words, the more patients want to delay surgery, the more they are prompted to receive multiple IA HA courses, even if the reduction in pain may not be sufficient as compared with what early TKA would achieve. The increased time from declared diagnosis of KOA to TKA observed for patients receiving IA HA injections might thus reflect not only a pharmacological effect of HA, but also the preferences of patients and/or physicians.

Among limitations, few publications reported confounding factors that could modify time to TKA, such as KOA severity, psycho-social context and comorbidities. The CCI did not differ in the study of Delbarre et al. [55]. In the study of Altman et al., it was significantly lower but not clinically relevant in the HA group compared to the control group (0.6 vs. 0.7, *p* < 0.001). In another study, the prevalences of a comorbidity were 61.6% and 33% in the HA and PRP groups, but both types of injections may be used to avoid TKA in patients with comorbidities, and the study had no group of patients without HA or PRP injections [60]. The other studies had no data on comorbidities or no group without any injection. These may be factors of the surgical decision as absolute or relative contraindications that may delay the surgical procedure. It could constitute a bias of indication for IA injections in the studies. In addition, the CCI does not include BMI or body mass, which are important factors to consider for TKA indication. Risk of surgical revision increases with BMI [66]: with BMI > 30 kg/m^2^, surgical complications increase 3% [67]. In the meta-analysis, unfortunately only a few studies mentioned BMI.

Second, the results were heterogeneous, reflecting the lack of consensus in the use of IA HA injections and practices among countries. For example, studies included patients with different clinical or structural disease severity (different VAS pain, KL stage, function score). The onset of KOA symptoms or KOA diagnosis (especially based on codes) is not necessarily the disease onset. However, the research was exhaustive by including all articles reporting a delay to TKA. There is also a lack of data for statistical analyses. Results of some studies were not pooled in the meta-analysis because the results overlapped with those of other studies in that they used the same database [39,50], or because the studies lacked quantitative data (number of patients, mean delay, SD, control group, data on TKA, etc.). However, the data available were used maximally for the analyses, and authors of selected studies were contacted (without success) to obtain supplementary data. Despite a growing interest in PRP injection use in OA, data on PRP injection and the TKA requirement remain scarce. Another limitation is the possible publication bias, which could not be analyzed by a funnel plot.

Despite these limitations, the study has several strengths. This is the first meta-analysis on the effect of IA HA or PRP injection on time to TKA. The research has been exhaustive and included good-quality studies (mean STROBE score 63%) with more than 2,820,000 patients. All possible time outcomes (mean delay, prevalence) and all possible time points (from KOA diagnosis in the database and from IA injection to TKA) were evaluated to fully exploit the data. The main outcome is original.

In conclusion, the results suggest that IA HA injections are associated with a 10-month delay in TKA in KOA. Causality cannot be concluded because of confounding factors and indication bias. Data were insufficient to conclude on any effect of PRP injections on TKA delay. Further research is needed to address this critical issue in the management of KOA.

## Figures and Tables

**Table 1:** Characteristics of included studies.

Author and Year of Publication	Quality Score STROBE (%)	Patients with IA Injection (*n*) (Population)	Injection Products (Intervention)	Patient without IA Injection (*n*) (Comparator)	Data of Interest Available (Outcome)			
					Time from KOA Diagnosis to TKA	Prevalence of TKA from Diagnosis	Time from IA Injection to TKA	Prevalence of TKA from IA Injection
Altman-dasa, 2015 [38]	65%	50,349	All HA	131,673	Yes	Yes	No	No
Delbarre, 2017 [55]	81%	1296	All HA	366	Yes	Yes	No	No
Ong, 2016 [39]	76%	9586	All HA	25,560	Yes	No	No	No
Altman-kim, 2015 [37]	76%	8423	All HA	14,132	Yes	No	No	No
Ong, 2019a [41] *	66%	88,501	All HA	1,941,996	No	Yes	No	No
Ong, 2019b [40] *	76%	37,160	All HA	104,145	Yes	Yes	No	No
Etter, 2020 [42]	77%	4376	HMW HA	90,316	Yes	No	No	No
Abbott, 2013 [51]	Abstract	6981	All HA	19,627	Yes	No	No	No
Malanga, 2020 [50]	68%	45,801	HA	229,455	Yes	No	Yes	No
Latourte, 2022 [57]	Cohort	191	All HA	465	Yes	No	No	No
Korzmaz, 2013 [58]	44%	197	LMW HA	487	No	No	No	Yes
Jurado, 2012 [53]	61%	202	HMW HA	22	No	No	Yes	No
Dasa, 2018 [39]	68%	50,389	HA	0	No	No	Yes	No
Waddell, 2014 [44]	67%	1342	HMW HA	0	No	No	Yes	Yes
Bowman, 2018 [45]	67%	120	HMW HA	0	No	No	Yes	Yes
Miler, 2017 [47]	69%	218	HMW HA	0	No	No	No	Yes
Turajane, 2008 [59]	44%	183	LMW HA	0	No	No	Yes	Yes
Lundstrom, 2019 [46]	53%	1147	LMW HA	0	No	No	No	Yes
Anand, 2018 [48]	55%	130	HMW HA	0	No	No	No	Yes
Barrett, 2002 [49]	67%	176	LMW HA	0	No	No	No	Yes
Whitman, 2010 [62]	27%	220	HMW HA	0	No	No	No	Yes
Campbell, 2004 [61]	29%	61	HMW HA	0	No	No	No	Yes
Evanich, 2001 [52]	67%	70	HMW HA	0	No	No	Yes	Yes
Mazieres, 2007 [56]	67%	296	HMW HA	0	No	No	No	Yes
Annaniemi, 2019 [60]	59%	8694	All HA PRP	0	No	No	No	Yes
Sanchez, 2020 [54]	67%	186	PRP	0	No	No	Yes	Yes

* Same study with different analysis for each article. CS = corticosteroids; HA = hyaluronic acid; HMW = high molecular weight, IA = intra-articular; TKA = total knee arthroplasty; KOA = knee osteoarthritis; LMW = low molecular weight, PRP = platelet-rich plasma.

**Table 2:** Characteristics of studies and included patients.

Author and Year of Publication	Patients (*n*)	Database/Years of Inclusion	OA Diagnostic Criteria	With TKA	Without TKA	Age (years), Mean ± SD	Female (%)	Mean BMI (kg/m²)
Altman-dasa, 2015 [38]	182,022	IMS Health Database/ 2007–2013	Codes	Yes	No	61 ± 8.9	58.7%	-
Delbarre, 2017 [55]	14,782	French medical insurance/ 2006–3013	X-ray images of the knee followed by an IA injection, prescribed by an OA specialist	Yes	Yes	67.67 ± 10.41	66%	-
Ong, 2016 [39]	35,142	5% Medicare/ 2005–2012	OA knee or osteoarthritis with pain leg codes	Yes	No	-	61.2%	-
Altman-kim, 2015 [37]	22,555	Truven market scan commercial/ 2006–2011	Codes	Yes	No	-	61.7%	-
Ong, 2019a [41]	2,030,497	Optum informatics/ 2006–2016	Codes	Yes	Yes	-	-	-
Ong, 2019b [40]	141,305		Codes	Yes	No	-	-	-
Etter, 2020 [42]	30,028	Truven market scan commercial/ 2008–2017	Codes by an orthopedics	Yes	No	-	58.2%	-
Abbott, 2013 [51]	-	Truven market scan commercial/ 2007–2011	First visit to an OA specialist	Yes	No	-	-	-
Malanga, 2020 [50]	275,256	5% Medicare/2010–2015	Codes	Yes	Yes	-	-	-
Latourte, 2022 [57]	656	No/2013	ACR	Yes	Yes	62.21 ± 8.45	70.3%	30.3 ± 6.2
Korzmaz, 2013 [58]	684	No/2007–2009	NA	Yes	Yes	55.1 ± 12.8	83.6%	-
Jurado, 2012 [53]	224	No/2006–2009	Spanish recommendations	Yes	Yes	-	67.9%	-
Dasa, 2018 [43]	50,389	IMS Health Database/2007–2010	First injection of HA	Yes	No	57.5 ± 10.5	59.9%	-
Waddell, 2014 [44]	1342	No/1997–2010	-	Yes	Yes	67.5 ± 10.1	60.2%	31.5 ± 7
Bowman, 2018 [45]	102	No/2013–2016	-	Yes	Yes	60.1 ± -	71.6%	33 ± -
Miller, 2017 [47]	218	No/NA	ACR	Yes	Yes	70.5 ± 9.2	46.3%	30.5 ± 6.9
Turajane, 2008 [59]	183	No/2001–2004	ACR	Yes	Yes	68.7 ± -	74.9%	25.1 ± -
Lundstrom, 2019 [46]	1147	No/2008–2014	-	Yes	Yes	62.2 ± 14	65.7%	25.21 ± -
Anand, 2018 [48]	130	No/1999–2003	-	Yes	Yes	-	57.7%	-
Barrett, 2002 [49]	376	No/-	ACR	Yes	Yes	72 ± 11	-	-
Whitman, 2010 [62]	220	No/-	-	Yes	Yes	-	74.5%	-
Campbell, 2004 [61]	61	No/-	X-ray images or arthroscopy	Yes	Yes	62.2 ± -	44.3%	55.7%
Evanich, 2001 [52]	70	No/1997	X-ray images	Yes	Yes	66 ± 14	61%	-
Annaniemi, 2019 [60]	180	No/2014–2017	Radiography	Yes	Yes	61.3 ± 8.8	60.3%	29.8 ± 4.8
Mazieres, 2007 [56]	296	No/2003–2004	ACR	Yes	Yes	69 ± 10	65%	28 ± 5
Sanchez, 2020 [54]	481	No/2014–2019	Radiography	Yes	Yes	63.9 ± -	49.3%	-
	186					63.9 ± 7.4	-	-

ACR = American College of Rheumatology; BMI = body mass index; IA = intra-articular; OA = osteoarthritis; SD = standard deviation; TKA = total knee arthroplasty; - = not applicable.

**Table 3:** Prevalence of TKA after IA injection.

		After IA HA Injection	After PRP Injection
6 months	Barett, 2002 [49] Mazieres, 2007 [56]	20.3% 0.7%	- -
8 months	Campbell, 2004 [61]	11.4% *	
10 months	Evanich, 2001 [52]	28.6% *	-
1 year	Whitman, 2010 [62] Korkmaz, 2013 [58] Miller, 2017 [47]	1% 3.2% 10.4%	- - -
17 months	Annaniemi, 2019 [60]	36% *	3.3% *
2 years	Miller, 2017 [47]	18%	-
27 months	Bowman, 2018 [45]	19.6%	-
3 years	Dasa, 2018 [43]	25.7%	-
3.5 years	Turajane, 2008 [59] Miller, 2017 [47]	25% 37.2% *	- -
5 years	Latourte, 2022 [57]	25.7%	-
6 years	Sanchez, 2020 [54]	-	9.4%
6.5/7 years	Lundstrom, 2019 [46]	50%	-
7.3 years	Sanchez, 2020 [54]	-	31.6%
8 years	Waddell, 2014 [44]	25%	-

* = Prevalence on mean follow-up. HA = hyaluronic acid; IA = intra-articular; PRP = platelet-rich plasma; TKA = total knee arthroplasty; - = not applicable.

## Data Availability

Data may be obtained upon request to corresponding author.

## Supplementary Materials

The following supporting information can be downloaded at: https://www.mdpi.com/article/10.3390/jcm11143985/s1. Table S1: Characteristics of injection products and control care for knee osteoarthritis.
