# Supplementary material for: Time to Total Knee Arthroplasty after Intra-Articular Hyaluronic Acid or Platelet-Rich Plasma Injections: A Systematic Literature Review and Meta-Analysis
Source: J Clin Med. 2022 Jul 9;11(14):3985. doi: 10.3390/jcm11143985 (PMC9322631; doi:10.3390/jcm11143985)
Supplement: Supplementary file 1 [file jcm-11-03985-s001.zip › jcm-1760821-supplementary.pdf]

Supplementary Materials:

Table S1. Characteristics of injection products and control care for knee osteoarthritis.

Author and Year of Publication	Product	Posology	Preparation of PRP	Control	Posology of the Control Product
Altman-dasa 2015 [38]	HA	-	-	Standard care	-
Delbarre 2017 [55]	HA	-	-	IA CS	-
Ong 2016 [39]	HA	-	-	Standard care	-
Altman-kim 2015 [37]	HA	-	-	Standard care	-
Ong 2019 a [41] *	HA	-	-	Standard care	-
Ong 2019 b [40] *	HA	-	-	Standard care	-
Etter 2020 [42]	Hylan GF 20 (Orthovisc)	-	-	Standard care	-
Abbott 2013 [51]	HA	-	-	Standard care	-
Malanga 2020 [50]	HA	-	-	Standard care	-
Latourte 2022 [57]	HA	-	-	Standard care	-
Korzmaz 2013 [58]	Adant	3 injections at 1-week interval	-	DICLOFENAC	75 mg × 2/day for 10 days
Jurado 2012 [53]	NASHA	-	-	Standard care	-
Dasa 2018 [39]	HA	-	-	No	-
Waddell 2014 [44]	Hylan GF 20	One injection 6 mL or 3 injections of 2 mL at 1-week interval	-	No	-
Bowman 2018 [45]	Sodium hyaluronate (Supartz, synvisc or euflexxa)	-	-	No	-
Miler 2017[47]	Sodium Hyaluronate (Hyalgan)	5 injections at 1-week interval	-	No	-
Turajane 2008 [59]	Sodium Hyaluronate (Hyalgan)	3 injections at 1-week interval	-	No	-
Lundstrom 2019 [46]	HA	-	-	No	-
Anand 2018 [48]	Sodium Hyaluronate	-	-	No	-
Barrett 2002 [49]	Sodium Hyaluronate	-	-	No	-
Whitman 2010 [62]	Supartz	-	-	No	-
Campbell 2004 [61]	Hylan GF-20	3 injections of 2 mL at 1-week interval	-	No	-
Evanich 2001 [52]	Hylan GF-20	3 injections of 2 mL at 1-week interval	-	No	-
Mazieres 2007 [56]	Sodium Hyaluronate (Suplasyn)	3 injections at 1-week interval	-	No	-
Annaniemi 2019 [60]	HA	1 injection or 3 injections at 1-week interval	Commercial glo PRP kit: 10 mL blood centrifuged 5 min at 1200 rpm/min	PRP	3 injections at 10-14 days' interval
Sanchez 2020 [54]	PRP	3 injections at 1-week interval or 1 IA injection with 2 intra-	9 mL blood centrifuged at 580 g for 8 min at room temperature	No	-

		osseous injection followed by IA injection 1 week later			

CS = corticosteroids; HA = hyaluronic acid; IA = intra-articular; NASHA = Non-animal stabilized hyaluronic acid stabilized, PRP = platelet-rich plasma; - = not applicable.
